# Comparative evaluation of optical properties of lithium disilicate crowns fabricated by pressable and CAD CAM methods – An in vitro study

**DOI:** 10.1016/j.jobcr.2025.08.001

**Published:** 2025-08-11

**Authors:** Shweta A. Gaikwad, Amit K. Jagtap, V.N.V. Madhav

**Affiliations:** Department of Prosthodontics, Crown & Bridge and Implantology, Dr. D. Y. Patil Dental College and Hospital, Dr. D. Y. Patil Vidyapeeth, Pimpri, Pune, 411018, India

**Keywords:** Lithium disilicate crowns, Pressable ceramics, Optical properties, Color difference, Shade matching

## Abstract

**Aim:**

The aim of this study was to compare the optical performance of pressable and milled lithium disilicate crowns, focusing on their shade-matching accuracy against the VITA Classical A1 shade guide.

**Material and methods:**

20 samples each of lithium disilicate crowns were fabricated using pressable and CAD/CAM-milled techniques and VITA A1 shade tab as the control. A spectrophotometer was used to measure the optical properties of the crown, namely, brightness (L∗), red-green (a∗), blue-yellow (b∗), and color difference (ΔE) values. Data obtained was analyzed statistically to assess shade-matching precision and optical differences.

**Results:**

The lightness (L∗) value of the pressable group (92.22 ± 2.67) was significantly higher than that of the milled group (88.10 ± 1.08) and the control group (87.78 ± 0.08). Regarding the red–green axis (a∗), the pressable group had the highest mean value (0.97 ± 0.37), followed by milled (0.85 ± 0.24) and control (0.35 ± 0.11), showed statistically significant differences. In terms of yellow-blue chroma (b), the pressable crowns recorded the highest value (14.89 ± 0.99), followed by the control (14.43 ± 0.07) and milled (13.56 ± 0.25). Milled crowns exhibited a significantly lower ΔE (1.49 ± 0.42) compared to pressable crowns (4.71 ± 2.55).

**Conclusion:**

Milled lithium disilicate crowns showed superior shade-matching accuracy, which exhibited lower color difference (ΔE) values, inferring closer color alignment with the A1 standard. Pressable crowns showed enhanced translucency and esthetic vibrancy as contributed by higher brightness and chromatic intensity, but exhibited greater variability in color matching.

## 1 Introduction

Ceramics are highly valued in restorative dentistry for their attractive appearance, compatibility with the body, and ability to mimic the look of natural teeth. Among the many ceramic materials available, lithium disilicate, particularly the IPS e.max system, has become a leading choice for esthetic restorations due to its exceptional traits, such as translucency and opalescence.^1^^,^^2^ The IPS e.max system features two materials: IPS e.max Press and IPS e.max CAD, each made using different fabrication methods that affect their visual properties.^3^ This study examines the optical qualities of crowns produced by these approaches, with a special focus on translucency and overall esthetic outcomes.

The pressable technique uses a heat-pressing approach, where pre-shaped IPS e.max Press, ingots made of lithium disilicate are heated to 850–950 °C and pushed into a mold with intense pressure. This allows the material to fill the mold exactly, replicating the tooth preparation's fine details for a tight fit.^4^

Once pressed, the restoration cools and is typically coated with feldspathic ceramic to adjust its shade and texture, resulting in a lifelike look. This technique excels at producing crowns with superb translucency and consistent color, making it well-suited for anterior teeth. It also ensures restorations have excellent edge precision and even material density, which enhances their durability and strength. However, the process involves significant hands-on work and time, which can complicate achieving uniform results and lengthen production.^5^, ^6^, ^7^

In contrast, the CAD/CAM milled method employs computer-aided design (CAD) and computer-aided manufacturing (CAM) to craft restorations from pre-formed blocks of lithium disilicate. It starts by scanning the prepared tooth to form a 3D digital model. Custom software designs the restoration, which a milling machine then sculpts into its final form. After milling, the piece is crystallized in a furnace to boost its durability, color, and clarity.^8^ This technique is streamlined, delivering accurate and repeatable results faster than the pressable approach. It ensures a consistent fit, cutting down on human error during production. Still, the visual traits of milled crowns, like translucency and light spread, can vary, sometimes leading to a less lifelike appearance compared to pressed restorations.^9^

Translucency parameter and contrast ratio measure how much light a material lets through, with greater translucency creating a more natural effect.^10^

However, visual perception of color in dental restorations is not determined by color differences (ΔE) alone. It is influenced by optical factors such as translucency, scattering, fluorescence, and surface gloss.^11^ These properties affect how materials interact with ambient light, influencing perceived color under different clinical conditions.^12^ Lithium disilicate ceramics are favoured for their enamel-like translucency, allowing them to blend more naturally with surrounding dentition.^13^

Lithium disilicate ceramics are especially valued for their ability to mimic natural tooth structure due to their balanced translucency and light-scattering behavior.^14^ Understanding these optical properties is crucial for achieving high esthetic outcomes in clinical practice. Furthermore, the CIE-LAB color system is used to quantify color differences (ΔE), providing an objective means of comparing the shade and overall esthetic match between natural teeth and restorations.^1^ A smaller ΔE value indicates a closer match, which is critical for achieving a seamless integration of the restoration with the natural dentition.

This study aims to assess the optical properties of lithium disilicate crowns fabricated by the pressable (IPS e.max Press) and milled methods (IPS e.max CAD), examining their translucency and color. By utilizing objective measurements such as CIE-LAB coordinates and ΔE values, this research will offer valuable insights into the esthetic performance of these two fabrication techniques. Understanding how each method affects the optical properties of restorations will assist clinicians in selecting the most suitable approach for creating esthetic, lifelike crowns that meet the individual needs of their patients. The objective of the study was to evaluate and compare optical properties of Lithium disilicate crowns, fabricated via pressable (IPS e.max Press, Ivoclar, Vivadent) and CAD/CAM-milled (IPS e.max CAD, Ivoclar, Vivadent) materials with a control shade tab of A-1 shade, in relation to their shade-matching accuracy.

## 2 Methodology

### 2.1 Tooth preparation and guide creation

**Fig. 1:**
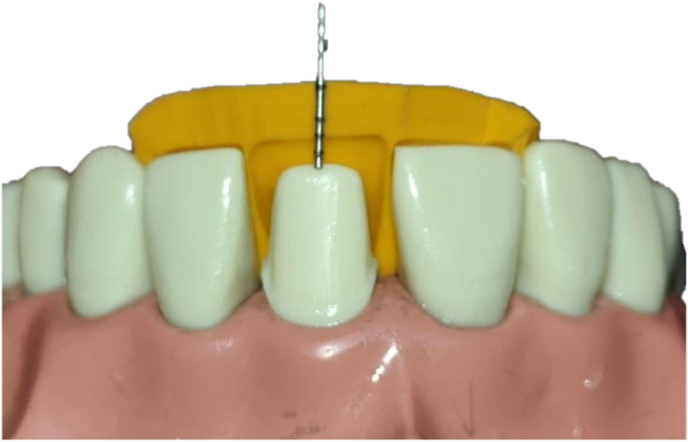
Preparation being verified with an index

A silicone putty index (3M ESPE, USA) of a maxillary central incisor typodont (Columbia) was made to guide tooth preparation, ensuring uniform reduction and crown thickness (Fig. 1). The preparation followed biomechanical standards: 2 mm incisal reduction, 1.5 mm labial/lingual reduction with a shoulder margin, 1.5 mm lingual clearance, and a 45° inciso-lingual bevel. The silicone index was used repeatedly to verify even reduction across specimens.

### 2.2 Digital scanning and crown design

The prepared tooth was scanned using an intraoral scanner (Trios 4, 3 shape, Copenhagen, Denmark) on a rotating platform, generating a 3D image. Using 3Shape software (3 Shape, Copenhagen, Denmark) a crown design was crafted and prepared for fabrication.

### 2.3 Specimen fabrication

**Fig. 2:**
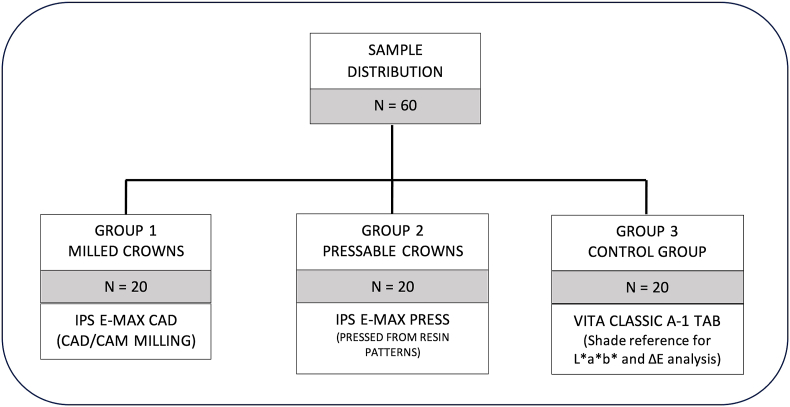
Different study groups

**Based on the sample size calculation, for the study groups, 20 samples for Group 1 (***Milled Crowns)***and Group 2***(Pressable Crowns)****,* were fabricated for the study.** And 20 readings of A-1 shade tab from the VITA Classical shade guide served as the control Group 3 (Fig. 2.).

*Group 1 (Milled Crowns):* The design was imported into Hyperdent software (FOLLOW-ME! Technology Systems, Germany). and sent to an Imes icore 150 ipro milling machine (imes-icore GmbH, Germany). IPS Empress CAD Multi A1/C14L ingots (Ivoclar Vivadent AG, Liechtenstein) were wet-milled into crowns. Post-milling, crowns were checked for fit, finished with sintered diamond tools and silicon polishers, and glazed with IPS e.max Ceram glaze paste (Ivoclar Vivadent AG, Liechtenstein) at 770 °C for 12 min in a Programmat EP5000 furnace(Ivoclar Vivadent AG, Liechtenstein).

*Group 2 (Pressable Crowns):* A Sprintray Pro 3D printer (SprintRay Inc., USA) created castable resin patterns from the digital design These were invested with IPS e.max Press powder (Pressvest, Ivoclar Vivadent AG) to form molds, burned out at 950 °C for 45 min in a UNIDENT furnace to remove resin, and pressed with IPS e.max Press A1 ingots in a Programmat EP5000 furnace (950 °C, 23 min). After cooling, crowns were divested, sandblasted with 110-μm Al_2_O_3_ at 4 bar, treated with INVEX liquid for 30 min, and sandblasted again. They were then fit-checked, finished with sintered diamonds, polished with silicon polishers, and glazed at 770 °C for 12 min. Crowns were cleaned with distilled water, air-dried, and stored in screw-top vials at room temperature.

*Group 3 (Control):* An A-1 shade tab from the VITA Classical shade guide served as the control. 20 readings were taken for the same.

### 2.4 Optical property evaluation

**Fig. 3:**
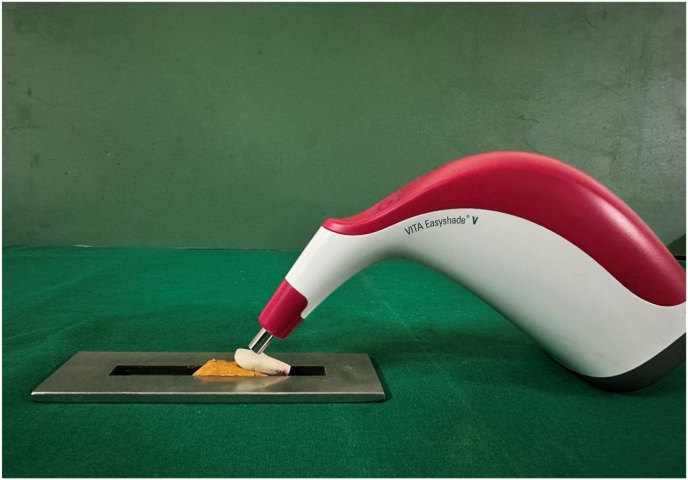
Spectrophotometer, testing the values.

**Fig. 4:**
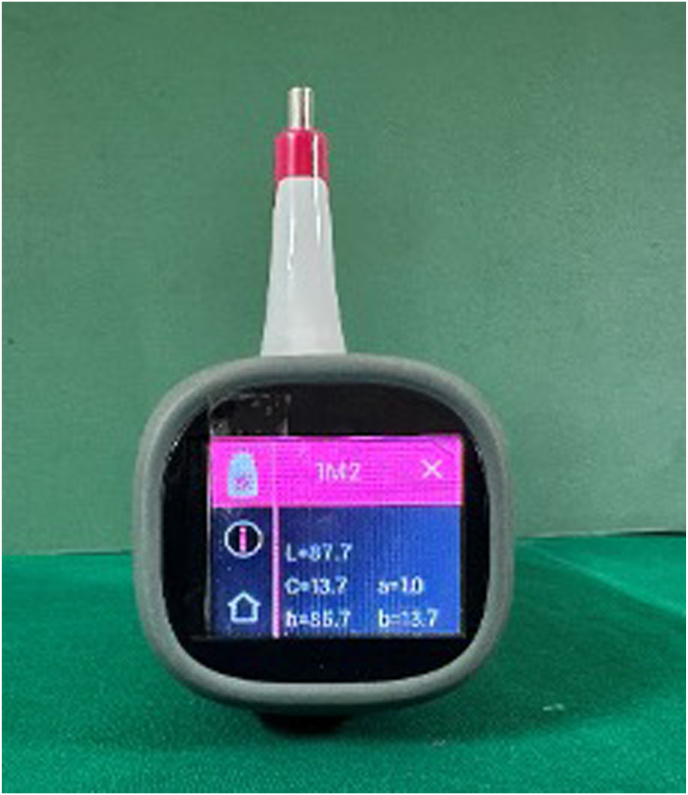
Spectrophotometer, displaying the values

CIE Lab values for the control were measured using the A1 shade tab. Well-fabricated crowns from both groups were seated on the prepared tooth, and their CIE Lab values were recorded at the middle third's center using a spectrophotometer. (VITA Easyshade V spectrophotometer (VITA Zahnfabrik, Germany) (Fig. 3, Fig. 4) The CIEDE2000 formula calculated ΔE_00_ color differences between crowns and the control.

• L = lightness (brightness)
• a = red-green axis
• b = blue-yellow axis

Color difference was computed as ΔE_00_ = [(ΔL)^2^ + (Δa)^2^ + (Δb)^2^]^1^/,^2^ with ΔL, Δa, and Δb as differences in respective axes.^15^ ΔE_00_ values were compared to the control to assess shade-matching accuracy of pressable and milled crowns. Results were analyzed to identify which method better matched the A1 shade, highlighting how material and fabrication affected optical and color properties. This approach ensured consistent preparation, crafting, and testing for a thorough comparison of pressable and milled lithium disilicate crowns’ shade accuracy and optics.

## 3 Results

Data were analyzed using one-way Analysis of Variance (ANOVA) followed by Tukey's HSD post hoc test to determine intergroup differences. Statistical significance was set at P < 0.05. All statistical analyses were performed using IBM SPSS Statistics for Windows, Version 27.0 (IBM Corp., Armonk, NY, USA). Statistical analysis was performed using IBM SPSS Statistics for Windows, Version 27.0 (IBM Corp., Armonk, NY, USA). Descriptive statistics including mean, standard deviation, minimum, and maximum values were computed for each group. To assess statistical differences among groups, one-way Analysis of Variance (ANOVA) was employed. ANOVA indicated significant differences, Tukey's Honest Significant Difference (HSD) test was applied for post hoc pairwise comparisons. A confidence interval of 95 % was used, and P < 0.05 was considered statistically significant. All statistical terms and abbreviations, including ΔE (color difference), L∗ (lightness), a∗ (red-green component), and b∗ (blue-yellow component), are defined in accordance with the CIE-LAB color system (Table 1).

**Table 1:** Mean ± SD and ANOVA results for optical properties and spectrophotometer scores.

Parameter	Group 1 (Milled, n = 20)	Group 2 (Pressable, n = 20)	Group 3 (Control, n = 20)	F-value	P-value
Lightness (L∗)	88.10 ± 1.08	92.22 ± 2.67	87.78 ± 0.08	43.979	<0.001
Red-Green (a∗)	0.85 ± 0.24	0.97 ± 0.38	0.35 ± 0.11	29.477	<0.001
Blue-Yellow (b∗)	13.56 ± 0.25	14.89 ± 0.99	14.43 ± 0.07	25.687	<0.001
Color Difference (ΔE)	1.49 ± 0.42	4.71 ± 2.55	–	–	<0.001

The optical properties of lithium disilicate crowns were compared across three groups—pressable, milled, and control (VITA A1 shade tab). All the specimens were analyzed with no losses or dropouts (Table 2).

**Table 2:** Tukey HSD post hoc comparisons of significant differences between groups.

Parameter	Comparison	Mean Difference	P-value
Lightness (L∗)	Control vs. Pressable	−4.44	<0.001
	Control vs. Milled	−0.32	0.817
	Pressable vs. Milled	4.12	<0.001
Red-Green (a∗)	Control vs. Pressable	−0.62	<0.001
	Control vs. Milled	−0.50	<0.001
	Pressable vs. Milled	0.12	0.347
Blue-Yellow (b∗)	Control vs. Pressable	−0.46	0.046
	Control vs. Milled	0.87	<0.001
	Pressable vs. Milled	1.33	<0.001

## 4 Discussion

This study assessed and compared the optical properties of lithium disilicate crowns fabricated via two different methods, pressable and CAD/CAM-milled methods by evaluating their CIELAB coordinates (L∗, a∗, b∗) and ΔE_00_ color differences which was compared with the VITA Classical A1 shade tab (control group). The results demonstrate that although both methods produced clinically acceptable crowns, milled crowns more closely matched the control group across most optical parameters, depicting higher shade-matching accuracy.

Lithium disilicate ceramics, have been widely accepted and favoured for their exceptional mechanical strength, optical behavior, and esthetic outcomes.^1^ Their unique microstructure, comprising a dense glass-ceramic matrix reinforced with lithium disilicate crystals, enables a harmonious balance between translucency and durability—making them highly suitable for both anterior and posterior restorations.^2^ The merits of lithium disilicate is that it can be made thin, without jeopardising the strength, and also offers excellent translucency and exceptional esthetic outcomes.^16^

The **fabrication technique** itself plays a vital role. In the pressable method, lithium disilicate ingots are heat-pressed into molds generated from wax patterns, allowing for anatomical detailing and layering but introducing variability due to manual finishing and multiple thermal cycles. These cycles may affect the microstructure and increase internal porosity, affecting light transmission and color saturation. However, CAD/CAM fabrication offers a subtractive method using homogenous blocks with tightly controlled composition, reducing variation and allowing precise replication of digital designs.^9^ In a research **Tuncel et al.** demonstrated, such control results in more consistent translucency values, mostly significant when multiple restorations are required in esthetically demanding zones.^17^

Moreover, sample handling during fabrication affects the optical performance of the final restoration. Even minor changes in glaze thickness, firing duration, or polish quality can significantly shift the final a∗ and b∗ values. **Barizon et al.** highlighted that glaze and staining protocols can affect the translucency parameter (TP) by altering the surface roughness and refractive index mismatch.^16^ Hence, despite similar materials being used, the fabrication process itself is a major contributing factor of final esthetic outcomes.

The results shows that while both fabrication techniques yield clinically acceptable esthetic outcomes, the **CAD/CAM-milled crowns** demonstrated significantly lower ΔE_00_ values and better shade-matching consistency. These results align with the findings of **Shirani et al.**, who reported that CAD/CAM lithium disilicate restorations produced more predictable color reproduction due to the standardized composition and homogeneity of pre-crystallized ingots.^18^ **Paravina et al.** also emphasized that clinical color matching depends not only on ΔE but also on translucency, material thickness, and the observer's perception.^11^

Also, **Heffernan et al.** observed that relative translucency varies among different ceramic systems on factors such as fabrication type and layer thickness, which directly influences L∗, a∗, and b∗ readings.^19^ In our study, **pressable crowns** exhibited higher brightness (L∗) and chromatic richness (a∗, b∗), likely due to the enhanced light scattering achieved through hand-layered feldspathic coatings and higher glass content. This is valuable in the anterior zone where esthetic vibrancy is prioritized. However, as supported by **Denry & Holloway**, the manual nature of pressing may introduce greater variability in shade outcome due to inconsistencies in pressure, firing, and layering.^2^

A gold standard method in quantifying optical behavior, is spectrophotometric evaluation as it offers repeatability and objectivity compared to visual methods.^20^^,^^21^ and is used by many researchers as a reference tool in studies of color matching.^22^, ^23^, ^24^ Spectrophotometers capture the entire spectrum of reflected or transmitted light and translate it into tristimulus values. However, the final visual outcome is still influenced by environmental factors such as light source, background shade, and translucency gradient, which underscores the importance of clinical context when interpreting ΔE values. As stated by **Vichi et al.**, high translucency can be a double-edged sword—improving esthetics but potentially revealing underlying discolorations or luting agents.^10^

The lightness (L∗) value of the pressable group (92.22 ± 2.67) was significantly higher than that of the milled group (88.10 ± 1.08) and the control group (87.78 ± 0.08). While higher lightness enhances esthetic vibrancy, it may lead to over-brightness, especially in posterior zones. The L∗ value of milled crowns was statistically closer to the control (p = 0.817), suggesting better integration with natural dentition. This finding is consistent with results reported by **Zarone et al**., who observed that milled lithium disilicate restorations fabricated from pre-crystallized blocks yield more predictable and controlled optical behavior.^8^ A research by **Ozturk et al**. observed higher L∗ values—in thicker lithium disilicate ceramics, reinforcing that lightness correlates strongly with material thickness and translucency.^25^

Regarding the **red–green axis (a∗),** the pressable group had the highest mean value (0.97 ± 0.37), followed by milled (0.85 ± 0.24) and control (0.35 ± 0.11), showed statistically significant differences. Although higher a∗ values can give a restoration a warm, reddish hue desirable for anterior esthetics, excessive redness (>+1.5) can appear unnatural. The **milled crowns again showed closer alignment with the control a∗** values (p = 0.347), indicating their superior chromatic neutrality. According to Yu and Lee, optimal a∗ values for natural teeth generally fall between +0.5 and + 1.5, with values above +2 being perceptible as too red.^26^

In terms of **yellow-blue chroma (b),** the pressable crowns recorded the highest value (14.89 ± 0.99), followed by the control (14.43 ± 0.07) and milled (13.56 ± 0.25). Although this elevated b∗ suggests a warm, lively tone beneficial for anterior teeth, **milled crowns were statistically closer to the control** (p < 0.001), making them more suitable when accurate shade replication is prioritized. Tuncel et al. noted that translucency enhances b∗ by allowing more light scattering, yet excessive yellowness may diverge from the desired natural shade in posterior restorations.^17^

The most definitive parameter, ΔE_00_, reflects the overall perceptible color difference from the control. Milled crowns exhibited a significantly lower ΔE (1.49 ± 0.42) compared to pressable crowns (4.71 ± 2.55). A ΔE_00_ below 2.25 is widely accepted as clinically imperceptible by most observers.^16^ Therefore, milled crowns displayed superior shade-matching accuracy in comparison to pressable crowns. These results align with the findings of Shirani et al., who reported better ΔE consistency in CAD/CAM lithium disilicate restorations compared to heat-pressed alternatives due to minimized human error and improved pigment homogeneity.^18^

### 4.1 Strengths of the study

The study has the following strengths, which is worth mentioning.

• The study employed objective spectrophotometric analysis (CIE L∗, a∗, b∗, and ΔE) to deliver measurable, repeatable data on the shade-matching capabilities of lithium di-silicate crowns.
• Milled crowns demonstrated lab values and ΔE closest to the VITA A1 control, establishing exceptional color accuracy, which is critical for posterior restorations.
• By incorporating both pressable and CAD/CAM fabrication methods under standardized conditions, the study enabled a direct and equitable comparison of their optical properties.
• Pressable crowns displayed greater brightness and chromatic intensity, making them suitable for anterior regions where esthetic vibrancy is prioritized.
• The study's design reduced the variability by maintaining the uniformity in tooth preparation, digital workflows, and material handling, hence enhancing the dependability of the findings.

### 4.2 Limitations

The study has several limitations, which are acknowledged as under:

• A relatively small sample size (n = 20 per group) limits statistical generalization. Future studies with larger cohorts are recommended.
• Controlled laboratory settings may not fully replicate the complexities of the oral environment, such as salivary interactions and thermal cycling. Long-term in vivo evaluations are necessary.
• The focus on a single monolithic shade (A1) from the VITA Classic guide restricts the findings' applicability to a broader range of shades. Future research should explore multiple shades to understand variations in translucency and color stability.
• This study does not account for the long-term effects of wear and aging on color stability, which are critical for clinical applications.

## 5 Conclusion

This in vitro study compared the optical properties of pressable and CAD/CAM-milled lithium disilicate crowns in relation to their shade-matching accuracy to the VITA Classical A1 shade guide.

• Milled lithium disilicate crowns showed superior shade-matching accuracy, which exhibited lower color difference (ΔE) values, inferring closer color alignment with the A1 standard.
• Pressable crowns showed enhanced translucency and esthetic vibrancy as contributed by higher brightness and chromatic intensity, but exhibited greater variability in color matching.

Thus, based on the results of the study it can be concluded that, milled lithium disilicate crowns offer more expected and consistent shade matching results whereas, pressable lithium disilicate crowns provide greater optical similarity and may be preferable especially in anterior restorations, for high esthetic demands.

## Ethical approval

The study was approved by the Institutional Ethics Committee, Dr. D. Y. Patil Dental College and Hospital, Dr. D. Y. Patil Vidyapeeth, Pimpri, Pune.

## Consent

As the study was an in-vitro study, no consents were required

## Source of funding

This research did not receive any external financial support. All resources utilized were provided by the institutional infrastructure of Dr. D. Y. Patil Dental College and Hospital, Dr. D. Y. Patil Vidyapeeth, Pimpri, Pune.

## Declaration of competing interest

The authors declare that they have no known competing financial interests or personal relationships that could have appeared to influence the work reported in this paper.
